# St. Louis Medical Journal

**Published:** 1849-09

**Authors:** 


*St. Louis Med. Journal.*—We have omitted heretofore to state, that during the recent extensive conflagration at St. Louis, Mo., the printing office from which the Med. Journal was issued, was destroyed, and the work has been, in consequence, temporarily suspended, The Editors have given notice that arrangements have been entered into for its continuance after a short interval.

